# Supplementary material for: Survive and Thrive: Outcomes of Children Enrolled in a Follow-Up Clinic for Small and Sick Newborns in Rural Rwanda
Source: Healthcare (Basel). 2024 Nov 26;12(23):2368. doi: 10.3390/healthcare12232368 (PMC11641708; doi:10.3390/healthcare12232368)
Supplement: Supplementary file 1 [file healthcare-12-02368-s001.zip › healthcare-3239453-supplementary.pdf]

Supplementary Materials

Table S1: Outcomes among observed children, excluding children with Hypoxic-Ischemic Encephalopathy (HIE)

Variable	Kayonza District N=351			Kirehe District N=335			All N=686		
	**Historical control**	**PDC Intervention**	**p-value**	**Historical control**	**PDC Intervention**	**p-value**	**Historical control**	**PDC Intervention**	**p-value**
Death, N=518, n (%)			0.007			0.026			<0.001
No	77 (90.6)	169 (98.3)		84 (83.2)	148 (92.5)		161 (86.6)	317 (95.5)	
Yes	8 (9.4)	3 (1.7)		17 (16.8)	12 (7.5)		25 (13.4)	15 (4.5)	
Stunting, N=474[1], n (%)			0.048			0.889			0.113
No	23 (30.7)	75 (44.6)		32 (38.1)	58 (39.5)		55 (34.6)	133 (42.2)	
Yes	52 (69.3)	93 (55.4)		52 (61.9)	89 (60.5)		104 (65.4)	182 (57.8)	
Underweight, N=477[1], n (%)			0.178			0.879			0.286
No	48 (63.2)	122 (72.2)		60 (71.4)	108 (73.0)		108 (67.5)	230 (72.6)	
Yes	28 (36.8)	47 (27.8)		24 (28.6)	40 (27.0)		52 (32.5)	87 (27.4)	
Wasting, N=467[1], n (%)			0.621			0.707			0.678
No	65 (90.3)	151 (92.1)		81 (96.4)	143 (97.3)		146 (93.6)	294 (94.5)	
Yes	7 (9.7)	13 (7.9)		3 (3.6)	4 (2.7)		10 (6.4)	17 (5.5)	
ASQ-3 total, N=470[1], n (%)			<0.001			0.003			0.095
Typical	5 (6.6)	64 (39.0)		28 (33.7)	23 (15.7)		33 (20.8)	87 (28.0)	
Potential delay	71 (93.4)	100 (61.0)		55 (66.3)	124 (84.3)		126 (79.2)	224 (72.0)	
**Anthropometric z-scores[2]**									
Height-for-age, N=474, median [IQR]	-2.61 [-3.44, -1.79]	-2.19 [-2.99, -1.29]	0.005	-2.36 [-3.00, -1.57]	-2.34 [-3.10, -1.43]	0.880	**-2.52 [-3.21, -1.71]**	**-2.23 [-3.04, -1.36]**	**0.064**
Weight-for-age, N=477, median [IQR]	-1.40 [-2.37, -0.54]	-1.31 [-2.15, -0.57]	0.430	-1.57 [-2.24, -0.78]	-1.41 [-2.05, -0.57]	0.576	-1.43 [-2.30, -0.63]	-1.33 [-2.10, -0.57]	0.316
Weight-for-height, N=467, median [IQR]	0.01 [-0.93, 0.61]	-0.18 [-1.11, 0.39]	0.319	-0.13 [-0.89, 0.56]	-0.22 [-0.83, 0.51]	0.950	-0.07 [-0.90, 0.58]	-0.19 [-0.92, 0.43]	0.480
**ASQ-3 z-scores by domain[3]**									
Fine Motor, N=459, median [IQR]	-2.68 [-3.10, -1.79]	-1.33 [-2.06, -0.34]	<0.001	-1.77 [-3.14, -0.89]	-1.70 [-2.78, -0.97]	0.339	-2.42 [-3.10, -1.32]	-1.41 [-2.40, -0.65]	<0.001
Gross Motor, N=475, median [IQR]	-2.09 [-3.65, -1.06]	-1.15 [-2.03, 0.03]	<0.001	-1.58 [-3.63, -0.21]	-0.80 [-2.57, 0.08]	0.035	-1.90 [-3.63, -0.64]	-0.95 [-2.21, 0.08]	<0.001
Communication, N=475, median [IQR]	-2.24 [-3.24, -1.59]	-1.38 [-2.61, -0.13]	<0.001	-1.54 [-3.26, -0.42]	-2.22 [-3.25, -0.82]	0.169	-1.96 [-3.26, -0.82]	-1.91 [-2.73, -0.42]	0.052
Problem solving, N=472, median [IQR]	-1.80 [-3.07, -0.64]	-0.51 [-1.86, 0.25]	<0.001	-0.26 [-1.26, 0.28]	-1.26 [-2.13, -0.44]	<0.001	-0.93 [-2.24, 0.08]	-0.93 [-1.97, -0.05]	0.898
Personal social, N=554, median [IQR]	-1.81 [-3.17, -0.77]	-0.65 [-1.48, 0.34]	<0.001	-1.60 [-2.45, -0.70]	-0.95 [-2.05, -0.23]	0.015	-1.62 [-2.73, -0.73]	-0.73 [-1.62, 0.06]	<0.001

CREDI[4] Overall score, median [IQR], N=211	-	-	-	51.58 [51.30, 51.95]	50.96 [50.40, 51.49]	<0.001	51.58 [51.30, 51.95]	50.96 [50.40, 51.49]	<0.001

PDC, Pediatric Development Clinic; **IQR**, interquartile range

[1]Only 476 children who participated in the homebased survey were eligible for analysis. An additional 9 children were missing data on stunting (4 with biologically implausible height-for-age and 5 children with missing height). For underweight, 1 child had biologically implausible weight-for-age data. Seventeen children had missing data on wasting (12 children with biologically implausible weight-for-height and 5 with missing height. Eight children had missing data on ages and stages questionnaire (ASQ) total score (1 whose true age differed from the administered ASQ survey be more than 3 months and 7 children who completed less than 4 out of 6 questions on at least one ASQ sub-domain).

[2]Z-scores were calculated using the age-specific means and standard deviations from the WHO's Child Growth Standards

[3]Z-scores were calculated based on the means and standard deviations reported in study of typically developing South African and Zambian children [32]

[4] Caregiver Reported Early Development Index (CREDI) was used only in Kirehe as it was designed to be more culturally neutral assessment of child development, however it was not widely available at the time of the study in Kayonza.

Fisher's exact test was used for categorical variables, while Wilcoxon Rank Sum test was used for continuous variables

Table S2: Association between the Pediatric Development Clinic intervention and outcomes (categorical variables), modeled with inverse probability weights accounting for differential death and non-participation in surveys between the groups, excluding children with Hypoxic-Ischemic Encephalopathy (HIE)

**Outcome variable**	**Kayonza District**		**Kirehe District**		**All**	
**BINARY OUTOMES**	**aOR (95% CI)**	**p-value**	**aOR (95% CI)**	**p-value**	**aOR (95% CI)**	**p-value**
Death (N=518)[1]	0.12 (0.03, 0.50)	0.003	0.51 (0.16, 1.58)	0.244	0.36 (0.18, 0.74)	0.005
Stunting (N=471)[2]	0.52 (0.28, 0.98)	0.044	1.10 (0.52, 2.33)	0.802	0.77 (0.51, 1.17)	0.218
Underweight (N=474)[2]	0.76 (0.41, 1.39)	0.370	1.22 (0.54, 2.75)	0.627	0.74 (0.48, 1.13)	0.164
Wasting (N=464)[2]	1.25 (0.41, 3.84)	0.699	0.13 (0.01, 1.92)	0.136	0.86 (0.36, 2.05)	0.733
ASQ-3 Total (N=467)[2]	0.10 (0.04, 0.29)	<0.001	1.58 (0.69, 3.62)	0.284	0.47 (0.29, 0.77)	0.002
**CONTINUOUS OUTCOMES**	**β (95% CI)**	**p-value**	**β (95% CI)**	**p-value**	**β (95% CI)**	**p-value**
**Nutrition indicators**						
Height-for-age z-score (N=471)[2]	0.52 (0.19, 0.86)	0.002	-0.07 (-0.54, 0.40)	0.761	0.22 (-0.01, 0.46)	0.063
Weight-for-age z-score (N=474)[2]	0.12 (-0.24, 0.49)	0.511	0.01 (-0.41, 0.44)	0.948	0.16 (-0.08, 0.40)	0.190
Weight-for-height z-score (N=464)[2]	-0.27 (-0.61, 0.07)	0.123	0.13 (-0.28, 0.54)	0.527	0.03 (-0.20, 0.27)	0.784
**Development indicators**						
ASQ-3 Fine Motor z-score (N=456)[2]	1.09 (0.77, 1.42)	<0.001	-0.28 (-0.75, 0.19)	0.237	0.71 (0.45, 0.96)	<0.001
ASQ-3 Gross Motor z-score (N=472)[2]	1.25 (0.74, 1.75)	<0.001	0.23 (-0.56, 1.02)	0.573	0.83 (0.48, 1.18)	<0.001
ASQ-3 Communication z-score (N=472)[2]	0.80 (0.42, 1.18)	<0.001	-0.61 (-1.32, 0.10)	0.090	0.42 (0.10, 0.74)	0.010
ASQ-3 Problem Solving z-score (N=469)[2]	0.78 (0.44, 1.11)	<0.001	-0.76 (-1.17, -0.35)	<0.001	0.38 (0.14, 0.63)	0.002
ASQ-3 Personal Social z-score (N=472)[2]	1.10 (0.64, 1.57)	<0.001	0.21 (-0.32, 0.74)	0.429	0.96 (0.66, 1.27)	<0.001

CREDI Overall score (N=210)[2]	-	-	0.07 (-0.20, 0.33)	0.607	0.07 (-0.20, 0.33)	0.607

aOR, adjusted odds ratio; **CI**, confidence interval

[1]Model was adjusted for lost to follow-up using inverse probability calculated based on intervention status, district of residence, sex, birthweight, condition (small baby), expected age at the follow-up survey date and estimated distance from home village to the nearest health facility. A weighted logistic regression model was adjusted for the same covariates.

[2]Model was adjusted for using inverse probability weights calculated based on intervention status, district of residence, sex, birthweight, condition (small baby), expected age at the follow-up survey date and estimated distance from home village to the nearest health facility. A weighted logistic regression model (for binary outcomes) or linear regression model (for continuous outcomes) was adjusted for intervention status, district of residence, sex, birthweight, condition (small baby), expected age at the follow-up survey date, primary caregiver's education level and marital status.

Table S3: Association between the Pediatric Development Clinic intervention and outcomes modeled with and without inverse probability weights accounting for differential death and non-participation in surveys between the groups

Outcome variable	Model	Kirehe District		Kayonza District		All	
		aOR (95% CI)	p-value	aOR (95% CI)	p-value	aOR (95% CI)	p-value
Death (N=613)	Unweighted[1]	0.62 (0.27, 1.39)	0.241	0.13 (0.03, 0.53)	0.005	0.49 (0.27, 0.90)	0.022
	Weighted[2]	0.62 (0.25, 1.55)	0.309	0.12 (0.03, 0.50)	0.003	0.49 (0.26, 0.92)	0.025
Stunting (N=544)	Unweighted[3]	1.19 (0.62, 2.30)	0.594	0.53 (0.29, 0.97)	0.040	0.86 (0.58, 1.27)	0.455
	Weighted[4]	1.17 (0.62, 2.19)	0.624	0.52 (0.28, 0.98)	0.044	0.87 (0.59, 1.28)	0.465
Underweight (N=553)	Unweighted[3]	1.22 (0.63, 2.36)	0.548	0.77 (0.42, 1.42)	0.402	0.79 (0.53, 1.19)	0.257
	Weighted[4]	1.19 (0.61, 2.34)	0.605	0.76 (0.41, 1.39)	0.370	0.77 (0.52, 1.15)	0.209
Wasting (N=537)	Unweighted[3]	0.37 (0.08, 1.61)	0.185	1.16 (0.40, 3.37)	0.784	0.72 (0.34, 1.50)	0.375
	Weighted[4]	0.35 (0.07, 1.78)	0.207	1.25 (0.41, 3.84)	0.699	0.72 (0.35, 1.48)	0.377
ASQ Total (N=465)	Unweighted[3]	1.34 (0.62, 2.93)	0.459	0.11 (0.04, 0.28)	<0.001	0.48 (0.29, 0.79)	0.003
	Weighted[4]	1.31 (0.60, 2.85)	0.497	0.10 (0.04, 0.29)	<0.001	0.48 (0.30, 0.77)	0.002

aOR, adjusted odds ratio; **ASQ**, Ages and Stages Questionnaire-3; **CI**, confidence interval

[1]Model adjusted for sex, birthweight, condition (small baby/hypoxic-ischemic encephalopathy [HIE]), expected age at the follow-up survey date and estimated distance from home village to the nearest health facility. [2]To account for lost to follow-up inverse probability weights were calculated based on the intervention status, district of residence, sex, birthweight, condition (small baby/HIE), expected age at the follow-up survey date and estimated distance from home village to the nearest health facility. [3]Unweighted models were adjusted for sex, birthweight, condition (small baby/HIE), expected age at the follow-up survey date, primary caregiver's education level and marital status.[4] Model additionally adjusted for non-participation in surveys using inverse probability calculated based on intervention status, district of residence, sex, birthweight, condition (small baby/HIE), expected age at the follow-up survey date and estimated distance from home village to the nearest health facility. A weighted logistic regression model was adjusted for sex, birthweight, condition (small baby/HIE), expected age at the follow-up survey date, primary caregiver's education level and marital status.

Table S4: Association between the Pediatric Development Clinic intervention and continuous outcomes modeled with and without inverse probability weights accounting for differential death and non-participation in surveys between the groups

Outcome variable	Model	Kirehe District		Kayonza District		All	
		β (95% CI)	p-value	β (95% CI)	p-value	β (95% CI)	p-value
**Nutrition indicators**							
Height-for-age z-score (N=544)	Unweighted[1]	0.02 (-0.35, 0.39)	0.910	0.50 (0.17, 0.83)	0.003	0.21 (-0.01, 0.43)	0.066
	Weighted[2]	0.04 (-0.35, 0.42)	0.856	0.52 (0.19, 0.86)	0.002	0.22 (-0.001, 0.44)	0.051
Weight-for-age z-score (N=553)	Unweighted[1]	-0.04 (-0.41, 0.34)	0.850	0.11 (-0.24, 0.46)	0.528	0.11 (-0.12, 0.34)	0.345
	Weighted[2]	-0.03 (-0.41, 0.35)	0.885	0.12 (-0.24, 0.49)	0.511	0.12 (-0.11, 0.34)	0.306
Weight-for-height z-score (N=537)	Unweighted[1]	0.15 (-0.22, 0.53)	0.418	-0.26 (-0.60, 0.08)	0.129	0.04 (-0.18, 0.27)	0.704
	Weighted[2]	0.17 (-0.20, 0.54)	0.377	-0.27 (-0.61, 0.07)	0.123	0.05 (-0.17, 0.27)	0.664
**Development indicators**							
ASQ Fine Motor z-score (N=533)	Unweighted[1]	-0.14 (-0.51, 0.23)	0.457	1.08 (0.76, 1.40)	<0.001	0.69 (0.46, 0.91)	<0.001
	Weighted[2]	-0.16 (-0.55, 0.23)	0.416	1.09 (0.77, 1.42)	<0.001	0.68 (0.45, 0.92)	<0.001
ASQ Gross Motor z-score (N=551)	Unweighted[1]	-0.30 (-0.99, 0.39)	0.392	1.26 (0.80, 1.72)	<0.001	0.56 (0.18, 0.93)	0.004
	Weighted[2]	-0.39 (-1.18, 0.39)	0.326	1.25 (0.74, 1.75)	<0.001	0.51 (0.13, 0.89)	0.008
ASQ Communication z-score (N=551)	Unweighted[1]	-0.69 (-1.28, -0.11)	0.020	0.80 (0.40, 1.19)	<0.001	0.30 (-0.02, 0.63)	0.064
	Weighted[2]	-0.69 (-1.33, -0.04)	0.037	0.80 (0.42, 1.18)	<0.001	0.31 (-0.02, 0.63)	0.064
ASQ Problem Solving z-score (N=548)	Unweighted[1]	-0.83 (-1.22, -0.45)	<0.001	0.77 (0.43, 1.10)	<0.001	0.21 (-0.03, 0.45)	0.087
	Weighted[2]	-0.83 (-1.20, -0.45)	<0.001	0.78 (0.44, 1.11)	<0.001	0.21 (-0.03, 0.45)	0.081
ASQ Personal Social z-score (N=551)	Unweighted[1]	-0.07 (-0.53, 0.38)	0.752	1.11 (0.67, 1.54)	<0.001	0.80 (0.51, 1.09)	<0.001
	Weighted[2]	-0.14 (-0.63, 0.34)	0.557	1.10 (0.64, 1.57)	<0.001	0.77 (0.47, 1.07)	<0.001
CREDI Overall score (N=261)	Unweighted[1]	-0.03 (-0.27, 0.22)	0.831	-	-	-0.03 (-0.27, 0.22)	0.831
	Weighted[2]	-0.04 (-0.28, 0.21)	0.778	-	-	-0.04 (-0.28, 0.21)	0.778

ASQ, Ages and Stages Questionnaire-3; **CI**, confidence interval

[1]Unweighted models were adjusted for sex, birthweight, condition (small baby/hypoxic-ischemic encephalopathy [HIE]), expected age at the follow-up survey date, primary caregiver's education level and marital status. [2]Model additionally adjusted for non-participation in surveys (using inverse probability weights calculated based on intervention status, district of residence, sex, birthweight, condition (small baby/HIE), expected age at the follow-up survey date and estimated distance from home village to the nearest health facility.
